# Risk Factors for Deep Sternal Wound Infection after Off-Pump Coronary Artery Bypass Grafting: a Case-Control Study

**DOI:** 10.21470/1678-9741-2020-0444

**Published:** 2022

**Authors:** Soslan Enginoev, Arian Arjomandi Rad, Sergey Ekimov, Dmitry Kondrat’ev, Gasan Magomedov, Alan Amirhanov, Bashir Tsaroev, Alexander Ziankou, Anna Motreva, Igor Chernov, Dmitry Tarasov, Bakytbek Kadyraliev, Michel Pompeu B. O. Sá

**Affiliations:** 1 Department of Cardiac Surgery, Federal Center for Cardiovascular Surgery, Astrakhan, Russia.; 2 Astrakhan State Medical University, Astrakhan, Russia.; 3 Department of Medicine, Faculty of Medicine, Imperial College London, London, United Kingdom.; 4 S. G. Sukhanov Federal Center of Cardiovascular Surgery, Perm, Russia.; 5 Department of Cardiovascular Surgery at the Pronto Socorro Cardiológico de Pernambuco (PROCAPE), Recife, PE, Brazil.

**Keywords:** Coronary Artery Bypass, Off-Pump, Acute Coronary Syndrome, Sternum, Wound Infection, Lower Extremity

## Abstract

**Introduction:**

The objective of this study was to identify risk factors for deep sternal wound infection (DSWI) after off-pump coronary artery bypass (OPCAB) grafting surgery.

**Methods:**

A total of 8,442 patients undergoing OPCAB from April 1, 2009 to December 31, 2018 were retrospectively analyzed. A total of 956 were eventually enrolled on this study based on our exclusion criteria. All subjects were divided into two groups: group 1 (n=63) - DSWI; group 2 (n=893) - without DSWI. Patients were excluded if they had one of the following: acute coronary syndrome, conversion to OPCAB grafting surgery, redo procedure, concomitant cardiac surgery procedures.

**Results:**

The prevalence of body mass index (BMI) ≥40 kg/m^2^ (7.9% *vs*. 1.9%, respectively; *P*=0.01), lower extremity atherosclerotic artery disease (23.8% *vs.* 7.2%, respectively; *P*=0.001) and use of bilateral internal thoracic artery (19.5% *vs*. 2.5%, respectively; *P*=0.008) was significantly higher in patients with DSWI. The incidence of morbidities, including reoperation for bleeding (26.4% *vs*. 2.1%, respectively; *P*<0.001), stroke (4.8% *vs*. 0.8%, respectively; *P*=0.02), acute renal failure (7.9% *vs*. 0.8%, respectively; *P*=0.001), delirium (7.9% *vs*. 1.7%, respectively; *P*=0.008) and blood transfusion (30.6% *vs*. 9.8%, respectively; *P*<0.001) was significantly higher in patients with DSWI.

**Conclusions:**

A BMI of >40 kg/m^2^, lower extremity artery disease, use of bilateral internal thoracic artery (BITA) graft, postoperative stroke, sepsis, reoperation due to postoperative complications and blood product requirement significantly increased the risk of sternal infection after OPCAB.

**Table:** Abbreviations, acronyms & symbols

CABG	= Coronary artery bypass grafting
BITA	= Bilateral internal thoracic artery
BMI	= Body mass index
CI	= Confidence interval
DSWI	= Deep sternal wound infection
EACTS	= European Association of Cardio-Thoracic Surgery
ESC	= European Society of Cardiology
IMA	= Internal mammary artery
ITA	= Internal thoracic artery
LITA	= Left internal thoracic artery
ONCAB	= On-pump coronary artery bypass
OPCAB	= Off-pump coronary artery bypass
OR	= Odds ratio
SPSS	= Statistical Package for the Social Sciences

## INTRODUCTION

Deep sternal wound infection (DSWI) constitutes a serious complication after coronary artery bypass grafting (CABG) surgery with potentially devastating consequences for patients. Although median sternotomy is known to offer an excellent approach in CABG, the impact of DSWI on patient prognosis remains significant. The advances made in the field of prevention allowed the incidence of DSWI to decrease drastically in CABG operations. Nevertheless, studies reported an incidence ranging from 1.8% to 2.1% and from 1.3% to 2.4%^[1-4]^, which, albeit low, still constitutes a major burden when put into the scale of the global number of CABG operations carried out worldwide. Indeed, DSWI has been shown to lead to life-threatening complications linked with an increase in long- and short-term mortality, morbidity, cost of care, prolonged hospital stay and in-hospital mortality ranging from 7% to 35%^[5-8]^.

The literature has also reported numerous risk factors potentially contributing to the development of DSWI in CABG, including the use of the internal thoracic artery for revascularization, long operative time, reoperation, an excessive use of bone wax and electrocoagulation, peripheral mechanical ventilation and other patient-related immunosuppressive risk factors^[9,10]^. However, data regarding off-pump coronary artery bypass (OPCAB) grafting and DSWI outcomes remain limited, with very few studies considering the risk factors for DSWI development in OPCAB.

The aims of this study were to identify risk factors for sternal infection after OPCAB grafting. The understanding of DSWI in OPCAB continues to evolve and the relationship has not been established yet, with only a limited number of studies published on the subject.

## METHODS

### Population and Study Design

This study conforms to the ethical guidelines of the 1975 Declaration of Helsinki as reflected in the *a priori* approval by the local Ethics Committee of our institution. We performed a retrospective review of a total of 9,790 patients in our institution who underwent CABG between April 1, 2009 and December 31, 2018, of which 8,442 (86%) were OPCAB. The left internal thoracic artery (LITA) was harvested in 97%. Patients were excluded if they had one of the following: acute coronary syndrome; conversion to on-pump coronary artery bypass (ONCAB) grafting; redo procedure; concomitant cardiac surgery procedures. Sixty-three patients (0.75%) with deep sternal infection and 893 patients without DSWI after OPCAB were selected according to the study exclusion criteria. The mean patient age was 63±6.5 years. Patients were followed up based on the information available in their electronic medical records, as well as through telephone interviews. The minimal period of follow-up for all patients was until discharge or death.

Patients were divided into two groups: group 1 presenting with DSWI post-OPCAB (n=63), and group 2 without DSWI post-OPCAB (n=893). Baseline patient characteristics and operative data were collected and compared between both groups. Data collected as part of the institutional OPCAB database included detailed information on patients’ demographics, baseline clinical characteristics and their laboratory data, findings, intraoperative variables and postoperative outcomes.

Diagnosis of DSWI was defined according to the guidelines provided by the Centers for Disease Control and Prevention, with all patients meeting at least one of the following: 1. Organism isolated from swab culture of mediastinal tissue or fluid; 2. Mediastinitis evidence observed intraoperatively; 3. One of the symptoms including chest pain, fever (>38°C), sternal instability, and presence of a purulent discharge from the mediastinum or swab culture isolated from the mediastinum tissue or fluid.

### Surgical Procedures

All OPCAB procedures were performed in a standard manner through a median sternotomy. After sternotomy, some surgeons used wax for sternal bleeding. Internal thoracic artery (ITA) was generally harvested with an electrocautery scalpel. The method of harvesting ITA was pedicled or skeletonized. Distal anastomosis was usually performed with 8-0 polypropylene suture. Proximal anastomosis was performed with clamped aorta or “no-touch” aorta technique. After completion of proximal anastomosis, control flowmetry of bypasses, pericardiostomy of the left pleural cavity, installation of drains, osteosynthesis of the sternum, and layer-by-layer wound closure were done. Prior to closing the sternum, some surgeons used topical vancomycin.

### Statistical Analysis

Data were analyzed using IBM SPSS version 25 (IBM Corp., Chicago, Illinois, United States). We used the Kolmogorov-Smirnov test to prove data for normal distribution. Quantitative data are expressed as the mean and standard deviation for normally distributed variables, and as median and interquartile range for variables that are not normally distributed. Differences between groups were compared using Student’s t test for normally distributed continuous data, Mann-Whitney U test for nonnormally distributed continuous variables, and c2 test for categorical variables. Odds ratio (OR) and 95% confidence intervals (CI) were analyzed to compare pooled proportions of participants presenting with and without the investigated risk factors A *P*<0.05 was considered statistically significant.

## RESULTS

### Preoperative Characteristics

Study demographics and preoperative characteristics are provided in Table 1. Body mass index (BMI) ≥40 kg/m^2^ was significantly higher among patients in the DSWI group (7.9% *vs*. 1.9%, respectively; *P*=0.01). However, no statistical difference between group 1 and group 2 in mean BMI (31±3.1 *vs*. 31±4.2; *P*=0.1), BMI 30-34.9 kg/m^2^ (41.3% *vs*. 38.5%; *P*=0.6) and BMI 35-39.9 kg/m^2^ (12.7% *vs*. 12.6%; *P*=0.9) was found. Furthermore, no significant difference was found in laboratory findings between group 1 and group 2 in terms of creatinine (82±10 mmol/l *vs*. 92±20 mmol/l; *P*=0.06), mean HbA1c (7.9±1.5 *vs*. 7.6±1.5; *P*=0.8) and HbA1c ≥7.5 (55.6% *vs*. 59%; *P*=0.8). Lower extremity atherosclerotic arterial disease (23.8% *vs*. 7.2%, respectively; *P*=0.001) was significantly higher in patients with DSWI.

### Operative Outcomes

Table 2 shows the operative characteristics. The median operative time was the same in both groups (125 [110-143] min *vs*. 150 [128-170] min, respectively; *P*=0.6), but the number of patients with an operative time of more than four hours was significantly higher in the group with DSWI (3.9% *vs*. 1.4%, respectively; *P*<0.001). The use of bilateral internal thoracic artery (BITA) was also associated with significantly higher rates of DSWI (19.5% *vs*. 2.5%, respectively; *P*=0.008). The use of topical vancomycin for prevention of DSWI was twice as frequent in patients without DSWI (76.4% *vs*. 38.1%, respectively; *P*<0.001).

**Table 1:** Preoperative risk factors for sternal infection.

Characteristics	DSWI (n=63)	Without DSWI (n=893)	*P*-value
Age (years), mean±SD	62±7	63±6.1	0.9
Age ≥65 years, n (%)	22 (34.9%)	306 (43.3%)	0.9
Male gender, n (%)	54 (85.7%)	761 (85.2%)	0.9
Active smoking, n (%)	33 (52.4%)	373 (41.8%)	0.1
Diabetes mellitus, n (%)	19 (30.2%)	234 (26.2%)	0.5
Body mass index (kg/m^2^), mean±SD	31±3.1	31±4.2	0.1
BMI >40 kg/m^2^ n (%)	5 (7.9%)	17 (1.9%)	0.01
NYHA classification, n (%):			
I	22 (34.9%)	323 (36.2%)	
II	32 (50.8%)	406 (45.5%)	
III	9 (14.3%)	161 (18%)	0.7
IV	0 (0%)	3 (0.3%)	
COPD, n (%)	14 (22.2%)	123 (13.8%)	0.06
Carotid stenosis, n (%)	9 (14.3)	96 (10.8%)	0.38
Lower extremity atherosclerotic artery disease, n (%)	15 (23.8%)	64 (7.2%)	0.001
Stroke, n (%)	2 (3.2%)	15 (1.7%)	0.3

BMI=body mass index; COPD=chronic obstructive lung disease; HbA1c=glycated hemoglobin; NYHA=New York Heart Association; SD=standard deviation

**Table 2:** Perioperative risk factor for sternal infection.

Characteristics	DSWI (n=63)	Without DSWI (n=893)	*P*-value
BITA grafts, n (%)	6 (19.5%)	22 (2.5%)	0.008
Use of topical wax, n (%)	12 (19%)	103 (11.5%)	0.07
Use of topical vancomycin, n (%)	24 (38.1%)	682 (76.4%)	<0.001
Total time of surgery, (min), median (25 and 75 percentiles)	125 (110-143)	150 (128-170)	0.6
Operative time, hrs			
<2	12 (19.7%)	97 (10.9%)	
2-3	33 (54.1%)	638 (71.5%)	
3-4	13 (21.3%)	144 (16.1%)	<0.001
4-5	1 (1.6%)	11 (1.2%)	
>5	2 (3.3%)	2 (0.2%)	

BITA=bilateral internal thoracic artery

The causative pathogen of DSWI was isolated and identified in 87.3% (n=55) of patients in the DSWI group (group 1). The organisms identified include *Staphylococcus epidermidis* (n=11), *Staphylococcus aureus* (n=12), *Escherichia coli* (n=4), *Klebsiella pneumoniae* (n=1), *Klebsiella aerogenes* (n=4), *Pseudomonas aeruginosa* (n=4), *Acinetobacter baumannii* (n=3), *Enterobacter cloacae* (n=5), Candida albicans (n=2), *Proteus mirabilis* (n=3), *Proteus vulgaris* (n=3) and *Serratia marcescens* (n=3).

### Postoperative Outcomes

Table 3 shows the postoperative patient characteristics. The incidence of morbidities, including reoperation for bleeding, stroke, acute renal failure, delirium, blood and plasma transfusion was significantly higher in patients with DSWI. Patients with DSWI had a higher incidence of sepsis (3.2% *vs*. 0.1%, respectively; *P*=0.01) and tracheostomy (4.8% *vs*. 0%, respectively; *P*<0.001). The length of hospital stay was significantly longer in patients with DSWI (33 [32-87] days *vs*. 10 [9-13] days, respectively; *P*<0.001). The operative mortality rate in patients with DSWI was significantly higher than in patients without DSWI (4.8% *vs*. 0.1%, respectively; *P*=0.001).

**Table 3:** Postoperative risk factors for sternal infection.

Characteristics	DSWI (n=63)	Without DSWI (n=893)	*P*-value
Creatinine, median (25 and 75 percentiles)	86 (73-110)	104 (87-116)	0.18
Reoperation for postoperative complications	28 (26.4%)	19 (2.1%)	<0.001
Stroke, n (%)	3 (4.8%)	7 (0.8%)	0.02
Blood transfusion, n (%)	19 (30.6%)	87 (9.8%)	<0.001
Plasma transfusion, n (%)	6 (9.7%)	44 (4.9%)	0.1
Delirium, n (%)	5 (7.9%)	15 (1.7%)	0.008
Perioperative MI, n (%)	1 (1.6%)	4 (0.4%)	0.2
Sepsis, n (%)	2 (3.2%)	1 (0.1%)	0.01
ARF, n (%)	7 (7.9%)	7 (0.8%)	0.001
Tracheostomy, n (%)	3 (4.8%)	0 (0%)	<0.001
Hospital stay (days), median (25 and 75 percentiles)	33 (32-87)	10 (9-13)	<0.001
Mortality, n (%)	3 (4.8%)	1 (0.1%)	0.001

ARF=acute renal failure; MI=myocardial infarction

## DISCUSSION

While the published evidence around isolated CABG reports the prevalence of DSWI at 1.3% to 2.4%^[1-4]^, the prevalence of DSWI observed in our cohort was 0.75% of the total population (63 patients). The following findings could be explained in terms of our shorter operative achievement time, which resulted from the long-term experience of our center and surgeons in OPCAB, also leading to a low incidence of significant bleeding. Indeed, the high volume of off-pump operations (86% of total CABG operations at our center) and the individual surgeon’s expertise have been long reported to be determining factors in OPCAB outcomes^[11]^, supported by multiple trials and observational studies.

With regards to preoperative risk factors for DSWI in patients undergoing CABG, the literature reports a diverse and wide range of findings, including: gender, tobacco smoking, diabetes mellitus and obesity^[5-10]^. Indeed, in line with the published literature, our study found that obesity (BMI >40 kg/m^2^) is a significant preoperative risk factor for the development of DSWI. Moreover, lower extremity chronic atherosclerotic artery disease was also found as another preoperative factor contributing to DSWI development (Table 1). The following was also found by previous reports which have associated lower extremity chronic atherosclerotic artery disease with the development of DSWI after CABG^[12]^.

The results of our analysis of postoperative outcomes variables point out to the use of BITA grafts being associated with significantly higher rates of DSWI (Table 2). Although there could be several plausible explanations, internal mammary artery (IMA) is known to be a major blood supply source to the sternum, thus its utilization in OPCAB could lead to a consequent local hypoperfusion, leaving the site highly exposed to the potential risk of developing DSWI^[13]^. The following concerns regarding DSWI and BITA grafts in CABG were also reported with regards to diabetic patients by the European Association of Cardio-Thoracic Surgery (EACTS) and the European Society of Cardiology (ESC)^[14]^. However, it is true that there is still no consensus on the use of BITA grafts in OPCAB and the development of DSWI, with scarce and conflicting evidence published in the literature.

The association of BITA and increased DSWI was reported for the first time in 1990^[15]^. Thereafter, a vast plethora of studies obtained similar results linking BITA to an increased risk of all types of sternal infection, with a metanalysis by Dai et al.^[5]^ reporting an increase of up to 62% of sternal infection. These results are further supported by the Arterial Revascularisation Trial, which found sternal wound infections (3.5% *vs*. 1.9%; *P*=0.005) and sternal reconstruction need to be more elevated with BITA^[16]^.

A first set of studies on ONCAB and OPCAB reported the early outcomes of BITA graft use and illustrated satisfactory outcomes related to DSWI and operative mortality^[17]^. The researchers had reported data of 560 hospital compromising 14,249 operations with BITA harvesting (32.6% of them were isolated CABG^[17]^. The results of these large-scale studies and metanalysis frequently excluded OPCAB patients outcomes with regards to DSWI. However, similar results were found in a study on OPCAB patients, with BITA not associated with DSWI even in diabetic patients^[18]^. On the other hand, Nakano et al.^[19]^, in their analysis of 1,500 OPCAB patients found, similarly to our study, risk factors in OPCAB comparable to those of ONCAB, with BITA associated to DSWI.

**Table 4:** Univariate and multivariate logistic regression analysis showing parameters associated with DSWI after OPCAB.

Variables	Unadjusted odds ratio (95% CI)	*P*-value	Adjusted odds ratio (95% CI)	*P*-value
BMI >40 kg/m^2^	4.4 (1.5-12.3)	0.01		
Lower extremity artery disease	4 (2.1-7.6)	0.001	3.9 (1.7-8.5)	0.001
Use of BITA grafts	4.1 (1.6-10.7)	0.008	4,6 (1.4-14.6)	0.01
Use of topical vancomycin	0.19 (0.11-0.32)	< 0.001		
Reoperation for postoperative complications	16.5 (8.8-30)	<0.001	34.5 (14.05-84.9)	< 0.001
Stroke	6.3 (1.5-25)	0.02		
Blood transfusion	4 (2.2-7.3)	<0.001		
Plasma transfusion	5 (1.7-14.3)	0.008		
Sepsis	29 (2.6-327)	0.01		
ARF	10 (3.3-35.4)	0.001	10.3 (2.3-47.2)	0.002

ARF=acute renal failure; BITA=bilateral internal thoracic artery; BMI=body mass index; CI=confidence interval

Additionally, re-exploration has been postulated to increase mediastinal exposure to airborne, environmental pathogens, thus increasing a patient’s susceptibility to the development of sternal infection^[20]^. Moreover, the use of blood products may lead to host immunosuppression and increase the risk of sternal infection^[19]^. In agreement with the published literature on CABG, our data has found that tracheostomy, blood transfusion, acute renal failure, delirium, stroke after operation and reoperation for postoperative complications can lead to an increased incidence of sternal infection (Table 4).

The increased burden of DSWI and its impact on outcomes in OPCAB have been also shown in our study. Our results found that the DSWI group suffered from significantly higher sepsis, longer hospital stay and mortality when compared to the non-DSWI group (Table 3). Complications associated to DSWI have long been known in cardiac surgery as associated with morbidity, mortality and higher costs^[21]^.

### LIMITATIONS

This study is a retrospective, nonrandomized analysis from a single medical center. Clinical decisions were made in a non-blinded fashion.

## CONCLUSIONS

The incidence of DSWI after OPCAB is 0.75%. BMI >40 kg/m^2^, lower extremity artery disease, use of BITA grafts, stroke after operation, sepsis, reoperation for postoperative complications, and high blood products requirement significantly increase the risk of sternal infection after OPCAB.

**Table:** Authors' roles & responsibilities

SE	Substantial contributions to the conception or design of the work; or the acquisition, analysis or interpretation of data for the work; drafting the work or revising it critically for important intellectual content; final approval of the version to be published
AAR	Drafting the work or revising it critically for important intellectual content; final approval of the version to be published
SE	Substantial contributions to the conception or design of the work; or the acquisition, analysis or interpretation of data for the work; drafting the work or revising it critically for important intellectual content; final approval of the version to be published
DK	Substantial contributions to the conception or design of the work; or the acquisition, analysis or interpretation of data for the work; drafting the work or revising it critically for important intellectual content; final approval of the version to be published
GM	Substantial contributions to the conception or design of the work; or the acquisition, analysis or interpretation of data for the work; drafting the work or revising it critically for important intellectual content; final approval of the version to be published
AA	Substantial contributions to the conception or design of the work; or the acquisition, analysis or interpretation of data for the work; drafting the work or revising it critically for important intellectual content; final approval of the version to be published
BT	Substantial contributions to the conception or design of the work; or the acquisition, analysis or interpretation of data for the work; drafting the work or revising it critically for important intellectual content; final approval of the version to be published
AZ	Substantial contributions to the conception or design of the work; or the acquisition, analysis or interpretation of data for the work; drafting the work or revising it critically for important intellectual content; final approval of the version to be published
AM	Substantial contributions to the conception or design of the work; or the acquisition, analysis or interpretation of data for the work; drafting the work or revising it critically for important intellectual content; final approval of the version to be published
IC	Substantial contributions to the conception or design of the work; or the acquisition, analysis or interpretation of data for the work; drafting the work or revising it critically for important intellectual content; final approval of the version to be published
DT	Substantial contributions to the conception or design of the work; or the acquisition, analysis or interpretation of data for the work; drafting the work or revising it critically for important intellectual content; final approval of the version to be published
BK	Substantial contributions to the conception or design of the work; or the acquisition, analysis or interpretation of data for the work; drafting the work or revising it critically for important intellectual content; final approval of the version to be published
MPBOS	Substantial contributions to the conception or design of the work; or the acquisition, analysis or interpretation of data for the work; drafting the work or revising it critically for important intellectual content; final approval of the version to be published
